# Crystal structure of 6-de­oxy-α-l-psico­furan­ose

**DOI:** 10.1107/S2056989015022215

**Published:** 2015-11-28

**Authors:** Akihide Yoshihara, Tomohiko Ishii, Tatsuya Kamakura, Hiroaki Taguchi, Kazuhiro Fukada

**Affiliations:** a Rare Sugar Research Center, Kagawa University, 2393 Ikenobe, Kagawa 761-0795, Japan; b Department of Advanced Materials Science, Faculty of Engineering, Kagawa University, 2217-20 Hayashi-cho, Takamatsu, Kagawa 761-0396, Japan; c Department of Applied Biological Science, Faculty of Agriculture, Kagawa University, 2393 Ikenobe, Kagawa 761-0795, Japan

**Keywords:** crystal structure, hydrogen bonding, de­oxy compound, rare sugar

## Abstract

The title compound, C_6_H_12_O_5_, was crystallized from an aqueous solution of 6-de­oxy-l-psicose (6-de­oxy-l-allulose, (3*S*,4*S*,5*S*)-1,3,4,5-tetra­hydroxy­hexan-2-one), and the mol­ecule was confirmed as α-furan­ose with a ^3^ *T*_4_ (or *E*_4_) conformation, which is a predominant tautomer in solution. This five-membered furan­ose ring structure is the second example in the field of the 6-de­oxy-ketohexose family. The cell volume of the title compound [742.67 (7) Å^3^, *Z* = 4 at room temperature] is only 1.4% smaller than that of β-d-psico­pyran­ose, C_6_H_12_O_6_ (753.056 Å^3^, *Z* = 4 at room temperature).

## Related literature   

For the predominant tautomer, α-furan­ose, of 6-de­oxy-l-psicose in aqueous solution, see: Yoshihara *et al.* (2015 ▸). For the crystal structure of chiral β-d-psicose, see: Kwiecień *et al.* (2008 ▸); Fukada *et al.* (2010 ▸). For the crystal structure of racemic β-*D*,l-psicose, see: Ishii *et al.* (2015 ▸). For the synthesis of 6-de­oxy-l-psicose, see: Shompoosang *et al.* (2014 ▸). For the crystal structures of 6-de­oxy-α-l-sorbo­furan­ose and 6-de­oxy-α-d-sorbo­furan­ose, see: Swaminathan *et al.* (1979 ▸); Rao *et al.* (1981 ▸); Jones *et al.* (2006 ▸).
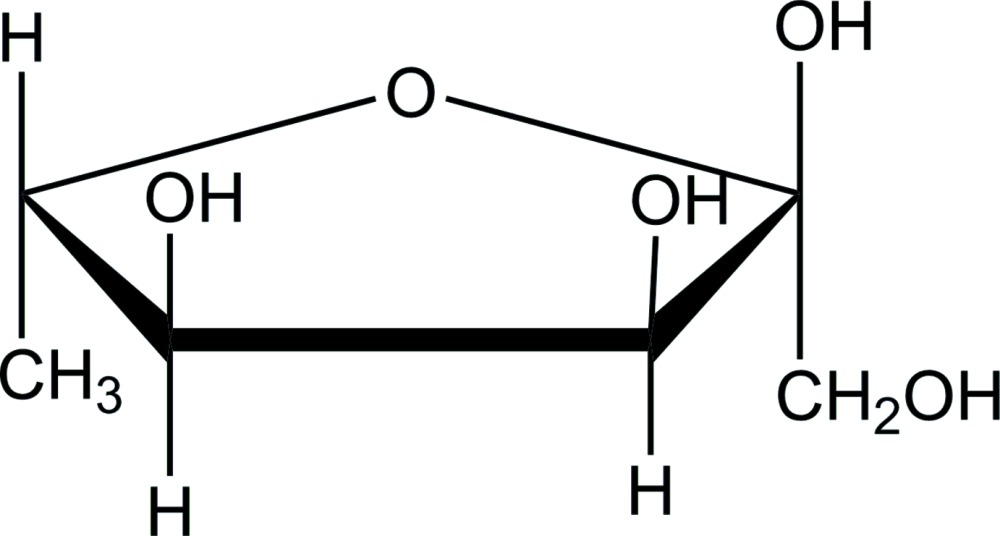


## Experimental   

### Crystal data  

C_6_H_12_O_5_
*M* *_r_* = 164.16
Orthorhombic, 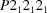

*a* = 5.7853 (3) Å
*b* = 8.9442 (5) Å
*c* = 14.3528 (8) Å
*V* = 742.69 (7) Å^3^
*Z* = 4
Cu *K*α radiation
μ = 1.12 mm^−1^
*T* = 296 K
0.10 × 0.10 × 0.10 mm

### Data collection  

Rigaku R-AXIS RAPID diffractometer
Absorption correction: multi-scan (*ABSCOR*; Higashi, 1995 ▸) *T*_min_ = 0.732, *T*_max_ = 0.894
13299 measured reflections
1358 independent reflections
1330 reflections with *F* ^2^ > 2σ(*F* ^2^)
*R*_int_ = 0.072

### Refinement  

*R*[*F* ^2^ > 2σ(*F* ^2^)] = 0.027
*wR*(*F* ^2^) = 0.065
*S* = 1.08
1358 reflections
105 parameters
H-atom parameters constrained
Δρ_max_ = 0.15 e Å^−3^
Δρ_min_ = −0.14 e Å^−3^
Absolute structure: Flack *x* determined using 521 quotients [(*I* ^+^)−(*I* ^−^)]/[(*I* ^+^)+(*I* ^−^)] (Parsons & Flack, 2004 ▸)
Absolute structure parameter: 0.03 (8)

### 

Data collection: *RAPID-AUTO* (Rigaku, 2009 ▸); cell refinement: *RAPID-AUTO*; data reduction: *RAPID-AUTO*; program(s) used to solve structure: *Il Milione* (Burla *et al.*, 2012 ▸); program(s) used to refine structure: *SHELXL2013* (Sheldrick, 2015 ▸); molecular graphics: *CrystalStructure* (Rigaku, 2014 ▸); software used to prepare material for publication: *CrystalStructure*.

## Supplementary Material

Crystal structure: contains datablock(s) global, I. DOI: 10.1107/S2056989015022215/is5433sup1.cif

Structure factors: contains datablock(s) I. DOI: 10.1107/S2056989015022215/is5433Isup2.hkl

Supporting information file. DOI: 10.1107/S2056989015022215/is5433Isup3.png

**Fig. 1.**
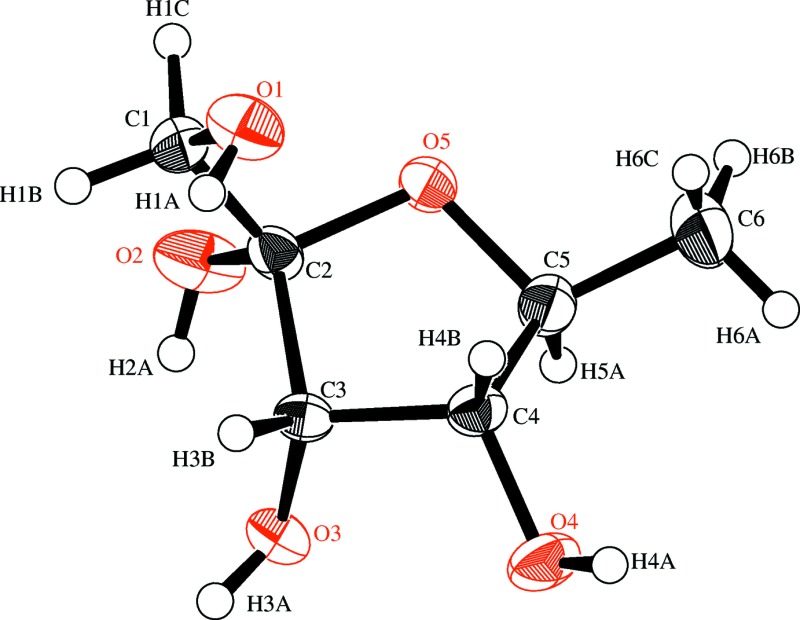
An ORTEP view of the title compound with the atom-labeling scheme. The thermal ellipsoids of all non-hydrogen atoms are drawn at the 50% probability level. H atoms are shown as small spheres of arbitrary radius.

**Fig. 2.**
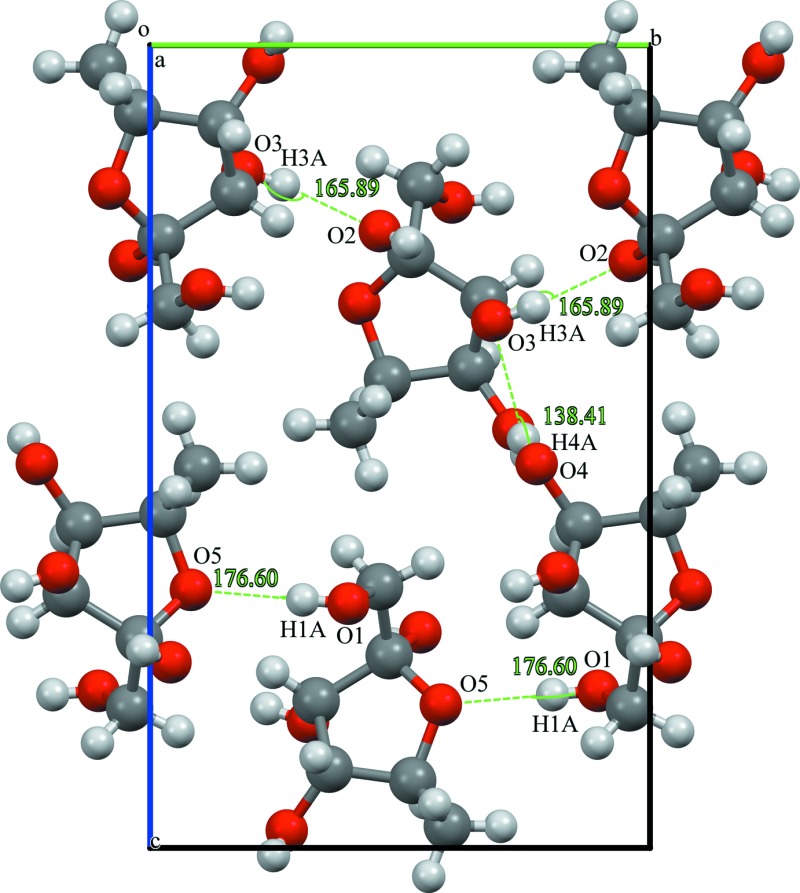
A packing diagram of the title compound viewed down the a-axis, showing the hydrogen-bonding network (green dashed lines).

ORTEP . DOI: 10.1107/S2056989015022215/is5433fig1.tif
An *ORTEP* view of the title compound with the atom-labeling scheme. The thermal ellipsoids of all non-hydrogen atoms are drawn at the 50% probability level. H atoms are shown as small spheres of arbitrary radius.

a . DOI: 10.1107/S2056989015022215/is5433fig2.tif
A packing diagram of the title compound viewed down the *a*-axis, showing the hydrogen-bonding network (green dashed lines).

CCDC reference: 1437931

Additional supporting information:  crystallographic information; 3D view; checkCIF report

## Figures and Tables

**Table 1:** Hydrogen-bond geometry (Å, °)

*D*—H⋯*A*	*D*—H	H⋯*A*	*D*⋯*A*	*D*—H⋯*A*
O1—H1*A*⋯O5^i^	0.82	2.02	2.839 (2)	177
O2—H2*A*⋯O1^ii^	0.82	2.13	2.819 (2)	142
O2—H2*A*⋯O3	0.82	2.08	2.592 (2)	121
O3—H3*A*⋯O2^iii^	0.82	1.93	2.732 (2)	166
O4—H4*A*⋯O3^iv^	0.82	2.24	2.902 (2)	138
O4—H4*A*⋯O4^iv^	0.82	2.26	2.987 (2)	148

Symmetry codes: (i) 
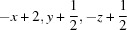
; (ii) 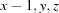; (iii) 
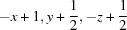
; (iv) 
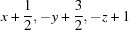
.

## supplementary crystallographic information

### S1. Comment

Psicose is classified into a rare sugar, and hardly exists in nature. In this study we prepare a single-crystal of 6-deoxy-*L*-psicose (Fig. 1), which is obtained by enzymatic isomerization of *L*-rhamnose, and investigate the structure by X-ray crystal analysis. The space group of this compound is orthorhombic *P*2_1_2_1_2_1_, which is the same as that of β-*D*-psicopyranose (*cf.* D-psicose; Kwiecień *et al.*, 2008; Fukada *et al.*, 2010). The molecular weight of 6-deoxy-*L*-psicose (C_6_H_12_O_5_; *m.w.* = 164.16) is about 10% smaller than that of *D*-psicose (180.16). On the other hand, the cell volume of 6-deoxy-α-*L*-psicofuranose is 742.67 (7) Å^3^ at r.t., which is a mere 1.4% smaller than that of β-*D*-psicopyranose (753.056 Å^3^ at r.t., *cf. D*-psicose; Kwiecień *et al.*, 2008; Fukada *et al.*, 2010). This imbalance of decreasing suggests that a weaker intermolecular interaction caused by a smaller molecular density can be expected. The melting point of 6-deoxy-α-*L*-psicofuranose has been observed to be 76 °C, which is about 30 °C lower than that of psicose (107.6 °C). This lower melting point is consistent with the suggested weaker intermolecular interaction.

We found that 6-deoxy-*L*-psicose molecules form a five-membered α-furanose ring structure in crystal. In the crystals of ketohexoses so far, six-membered pyranose ring structures have been mainly confirmed (*cf. D*-psicose; Kwiecień *et al.*, 2008; Fukada *et al.*, 2010, 1-deoxy-*L*-sorbose; Jones *et al.*, 2006). Because of the deoxygenation in the 6-deoxy-*L*-psicose molecule, the carbonyl group at the C-2 position cannot form hemiacetal with the C-6 but with the C-5 hydroxyl group. It should be noted that the crystal structure of 6-deoxy-*L*-sorbose, C-4 epimer of 6-deoxy-*L*-psicose, was reported to be α-furanose; C3'-*exo*-C4'-*endo*, _3_T^4^ (Swaminathan *et al.*, 1979). Therefore, the α-furanose structure observed in the crystal of 6-deoxy-*L*-psicose is the second example in 6-deoxy-ketohexose family, with ^3^T_4_ (or E_4_) conformation. An intramolecular hydrogen bond (O3—H3A···O5) has been observed both in a chiral *D*-psicose (Kwiecień *et al.*, 2008; Fukada *et al.*, 2010) and a racemic *D,*L**-psicose (Ishii *et al.*, 2015). This comes from two hydroxy groups located in a shorter distance from each other because of both axial conformations connecting to the C-3 and C-5 positions. On the other hand in the 6-deoxy-*L*-psicose, such an intramolecular hydrogen bond is not observed, because the hydroxy group at a C-5 position has been used for creating the ring structure. Intermolecular hydrogen bonds (O3—H3A···O2 and O1—H1A···O5) are also confirmed along the *b*-axis, and O4—H4A···O4 along the *a*-axis, as shown in Fig. 2.

### S2. Experimental

6-Deoxy-*L*-psicose was prepared from *L*-rhamnose by immobilized *L*-rhamnose isomerase and immobilized *D*-tagatose 3-epimerase in the batch reaction (Shompoosang *et al.*, 2014). After this reaction was reached equilibrium, the reaction mixture containing 6-deoxy-*L*-psicose was separated by column chromatography. The purified 6-deoxy-*L*-psicose solution was concentrated to 80% by evaporation. A seed crystal of 6-deoxy-*L*-psicose was added to the 80% 6-deoxy-*L*-psicose solution, which was kept at 30 °C. The tautomer ratio in aqueous solution at 30 °C is obtained as α-furanose: β-furanose: acyclic form = 72.9: 24.5: 2.69 (Yoshihara *et al.*, 2015). After one day, single crystals were obtained.

### S3. Refinement

H atoms bounded to methine-type C (H3B, H4B, H5A) were positioned geometrically and refined using a riding model with C—H = 0.98 Å and *U*_iso_(H) = 1.2*U*_eq_(C). H atoms bounded to methylene-type C (H1B, H1C) were positioned geometrically and refined using a riding model with C—H = 0.97 Å and *U*_iso_(H) = 1.2*U*_eq_(C). H atoms bounded to methyl-type C (H6A, H6B, H6C) were positioned geometrically and refined using a riding model with C—H = 0.96 Å and *U*_iso_(H) = 1.2*U*_eq_(C). H atoms bounded to O (H1A, H2A, H3A, H4A) were positioned geometrically and refined using a riding model with O—H = 0.82 Å and *U*_iso_(H) = 1.2*U*_eq_(O), allowing for free rotation of the OH groups.

### Crystal data

**Table:**

C_6_H_12_O_5_	*D*_x_ = 1.468 Mg m^−^^3^
*M**_r_* = 164.16	Cu *K*α radiation, λ = 1.54187 Å
Orthorhombic, *P*2_1_2_1_2_1_	Cell parameters from 7546 reflections
*a* = 5.7853 (3) Å	θ = 3.1–68.3°
*b* = 8.9442 (5) Å	µ = 1.12 mm^−^^1^
*c* = 14.3528 (8) Å	*T* = 296 K
*V* = 742.69 (7) Å^3^	Block, colorless
*Z* = 4	0.10 × 0.10 × 0.10 mm
*F*(000) = 352.00	

### Data collection

**Table:**

Rigaku R-AXIS RAPID diffractometer	1330 reflections with *F*^2^ > 2σ(*F*^2^)
Detector resolution: 10.000 pixels mm^-1^	*R*_int_ = 0.072
ω scans	θ_max_ = 68.2°, θ_min_ = 5.8°
Absorption correction: multi-scan (*ABSCOR*; Higashi, 1995)	*h* = −6→6
*T*_min_ = 0.732, *T*_max_ = 0.894	*k* = −10→10
13299 measured reflections	*l* = −17→17
1358 independent reflections	

### Refinement

**Table:**

Refinement on *F*^2^	H-atom parameters constrained
*R*[*F*^2^ > 2σ(*F*^2^)] = 0.027	*w* = 1/[σ^2^(*F*_o_^2^) + (0.0207*P*)^2^ + 0.1732*P*] where *P* = (*F*_o_^2^ + 2*F*_c_^2^)/3
*wR*(*F*^2^) = 0.065	(Δ/σ)_max_ < 0.001
*S* = 1.08	Δρ_max_ = 0.15 e Å^−^^3^
1358 reflections	Δρ_min_ = −0.14 e Å^−^^3^
105 parameters	Extinction correction: *SHELXL*
0 restraints	Extinction coefficient: 0.0144 (15)
Primary atom site location: structure-invariant direct methods	Absolute structure: Flack *x* determined using 521 quotients [(*I*^+^)-(*I*^-^)]/[(*I*^+^)+(*I*^-^)] (Parsons & Flack, 2004)
Secondary atom site location: difference Fourier map	Absolute structure parameter: 0.03 (8)
Hydrogen site location: inferred from neighbouring sites	

### Special details

**Table:**

Geometry. ENTER SPECIAL DETAILS OF THE MOLECULAR GEOMETRY
Refinement. Refinement was performed using all reflections. The weighted *R*-factor (*wR*) and goodness of fit (*S*) are based on *F*^2^. *R*-factor (gt) are based on *F*. The threshold expression of *F*^2^ > 2.0 σ(*F*^2^) is used only for calculating *R*-factor (gt).

### Fractional atomic coordinates and isotropic or equivalent isotropic displacement parameters (Å^2^)

**Table:**

	*x*	*y*	*z*	*U*_iso_*/*U*_eq_	
O1	1.1489 (3)	0.60359 (18)	0.19568 (11)	0.0370 (4)	
O2	0.5730 (3)	0.45936 (18)	0.23138 (13)	0.0419 (4)	
O3	0.5171 (2)	0.68432 (15)	0.34295 (10)	0.0274 (3)	
O4	0.8265 (3)	0.72675 (18)	0.47842 (10)	0.0371 (4)	
O5	0.8957 (3)	0.41852 (14)	0.32155 (9)	0.0297 (4)	
C1	0.9314 (4)	0.5411 (2)	0.17460 (13)	0.0299 (5)	
C2	0.7827 (3)	0.5218 (2)	0.26045 (13)	0.0239 (4)	
C3	0.7513 (3)	0.6680 (2)	0.31678 (13)	0.0217 (4)	
C4	0.9029 (3)	0.6417 (2)	0.40186 (13)	0.0243 (4)	
C5	0.8846 (4)	0.4748 (2)	0.41607 (13)	0.0288 (5)	
C6	1.0749 (5)	0.4055 (3)	0.47269 (18)	0.0472 (6)	
H1A	1.14196	0.69485	0.19094	0.0444*	
H1C	0.95355	0.44456	0.14516	0.0359*	
H1B	0.85181	0.60539	0.13061	0.0359*	
H2A	0.46647	0.5151	0.24595	0.0503*	
H3A	0.47011	0.76663	0.32637	0.0328*	
H3B	0.80479	0.75478	0.28114	0.0261*	
H4A	0.93817	0.75318	0.50951	0.0445*	
H4B	1.06309	0.66817	0.38712	0.0291*	
H5A	0.73436	0.45008	0.44361	0.0346*	
H6A	1.0678	0.44263	0.53538	0.0566*	
H6B	1.05675	0.29881	0.47311	0.0566*	
H6C	1.22165	0.4308	0.44577	0.0566*	

### Atomic displacement parameters (Å^2^)

**Table:**

	*U*^11^	*U*^22^	*U*^33^	*U*^12^	*U*^13^	*U*^23^
O1	0.0306 (8)	0.0317 (8)	0.0486 (9)	0.0022 (7)	0.0045 (7)	0.0026 (7)
O2	0.0265 (8)	0.0310 (9)	0.0683 (11)	−0.0008 (7)	−0.0091 (7)	−0.0203 (8)
O3	0.0221 (7)	0.0249 (8)	0.0351 (7)	0.0048 (6)	−0.0004 (6)	0.0008 (6)
O4	0.0320 (8)	0.0467 (10)	0.0325 (8)	0.0046 (8)	−0.0052 (6)	−0.0198 (7)
O5	0.0448 (9)	0.0177 (7)	0.0266 (7)	0.0073 (6)	−0.0022 (7)	0.0004 (5)
C1	0.0384 (11)	0.0263 (11)	0.0249 (10)	0.0058 (9)	−0.0033 (8)	−0.0040 (8)
C2	0.0267 (11)	0.0170 (9)	0.0282 (9)	0.0018 (8)	−0.0066 (8)	−0.0025 (7)
C3	0.0223 (9)	0.0165 (9)	0.0262 (9)	0.0002 (8)	0.0014 (8)	−0.0022 (7)
C4	0.0231 (9)	0.0244 (11)	0.0253 (9)	0.0001 (9)	−0.0000 (8)	−0.0055 (8)
C5	0.0333 (11)	0.0283 (11)	0.0248 (9)	0.0004 (9)	−0.0008 (8)	0.0004 (8)
C6	0.0607 (16)	0.0405 (14)	0.0404 (12)	0.0144 (12)	−0.0125 (12)	0.0049 (10)

### Geometric parameters (Å, º)

**Table:**

O1—C1	1.410 (3)	O2—H2A	0.820
O2—C2	1.399 (2)	O3—H3A	0.820
O3—C3	1.414 (2)	O4—H4A	0.820
O4—C4	1.408 (2)	C1—H1C	0.970
O5—C2	1.432 (2)	C1—H1B	0.970
O5—C5	1.448 (2)	C3—H3B	0.980
C1—C2	1.512 (3)	C4—H4B	0.980
C2—C3	1.548 (3)	C5—H5A	0.980
C3—C4	1.522 (3)	C6—H6A	0.960
C4—C5	1.510 (3)	C6—H6B	0.960
C5—C6	1.502 (3)	C6—H6C	0.960
O1—H1A	0.820		
			
C2—O5—C5	109.24 (14)	O1—C1—H1C	109.164
O1—C1—C2	112.20 (16)	O1—C1—H1B	109.167
O2—C2—O5	108.73 (15)	C2—C1—H1C	109.166
O2—C2—C1	107.19 (16)	C2—C1—H1B	109.170
O2—C2—C3	113.01 (16)	H1C—C1—H1B	107.872
O5—C2—C1	108.26 (16)	O3—C3—H3B	111.067
O5—C2—C3	106.18 (14)	C2—C3—H3B	111.068
C1—C2—C3	113.31 (16)	C4—C3—H3B	111.066
O3—C3—C2	109.81 (15)	O4—C4—H4B	109.543
O3—C3—C4	110.80 (15)	C3—C4—H4B	109.538
C2—C3—C4	102.75 (15)	C5—C4—H4B	109.537
O4—C4—C3	111.22 (16)	O5—C5—H5A	109.822
O4—C4—C5	114.02 (16)	C4—C5—H5A	109.816
C3—C4—C5	102.76 (15)	C6—C5—H5A	109.811
O5—C5—C4	102.34 (14)	C5—C6—H6A	109.470
O5—C5—C6	109.33 (18)	C5—C6—H6B	109.472
C4—C5—C6	115.43 (18)	C5—C6—H6C	109.471
C1—O1—H1A	109.468	H6A—C6—H6B	109.472
C2—O2—H2A	109.470	H6A—C6—H6C	109.470
C3—O3—H3A	109.471	H6B—C6—H6C	109.473
C4—O4—H4A	109.477		
			
C2—O5—C5—C4	−34.64 (18)	O5—C2—C3—C4	12.06 (17)
C2—O5—C5—C6	−157.50 (14)	C1—C2—C3—O3	135.42 (15)
C5—O5—C2—O2	−107.81 (16)	C1—C2—C3—C4	−106.65 (16)
C5—O5—C2—C1	136.05 (14)	O3—C3—C4—O4	−37.4 (2)
C5—O5—C2—C3	14.07 (18)	O3—C3—C4—C5	85.02 (16)
O1—C1—C2—O2	−179.95 (14)	C2—C3—C4—O4	−154.60 (14)
O1—C1—C2—O5	−62.8 (2)	C2—C3—C4—C5	−32.21 (16)
O1—C1—C2—C3	54.7 (2)	O4—C4—C5—O5	161.44 (14)
O2—C2—C3—O3	13.2 (2)	O4—C4—C5—C6	−79.9 (2)
O2—C2—C3—C4	131.17 (15)	C3—C4—C5—O5	40.95 (18)
O5—C2—C3—O3	−105.88 (16)	C3—C4—C5—C6	159.59 (14)

### Hydrogen-bond geometry (Å, º)

**Table:**

*D*—H···*A*	*D*—H	H···*A*	*D*···*A*	*D*—H···*A*
O1—H1*A*···O5^i^	0.82	2.02	2.839 (2)	177
O2—H2*A*···O1^ii^	0.82	2.13	2.819 (2)	142
O2—H2*A*···O3	0.82	2.08	2.592 (2)	121
O3—H3*A*···O2^iii^	0.82	1.93	2.732 (2)	166
O4—H4*A*···O3^iv^	0.82	2.24	2.902 (2)	138
O4—H4*A*···O4^iv^	0.82	2.26	2.987 (2)	148

Symmetry codes: (i) −*x*+2, *y*+1/2, −*z*+1/2; (ii) *x*−1, *y*, *z*; (iii) −*x*+1, *y*+1/2, −*z*+1/2; (iv) *x*+1/2, −*y*+3/2, −*z*+1.
