# Supplementary figures and images for: Crystal structure of 6-de­oxy-α-l-psico­furan­ose
Source: Acta Crystallogr E Crystallogr Commun. 2015 Nov 28;71(Pt 12):o993–4. doi: 10.1107/S2056989015022215 (PMC4719940; doi:10.1107/S2056989015022215)

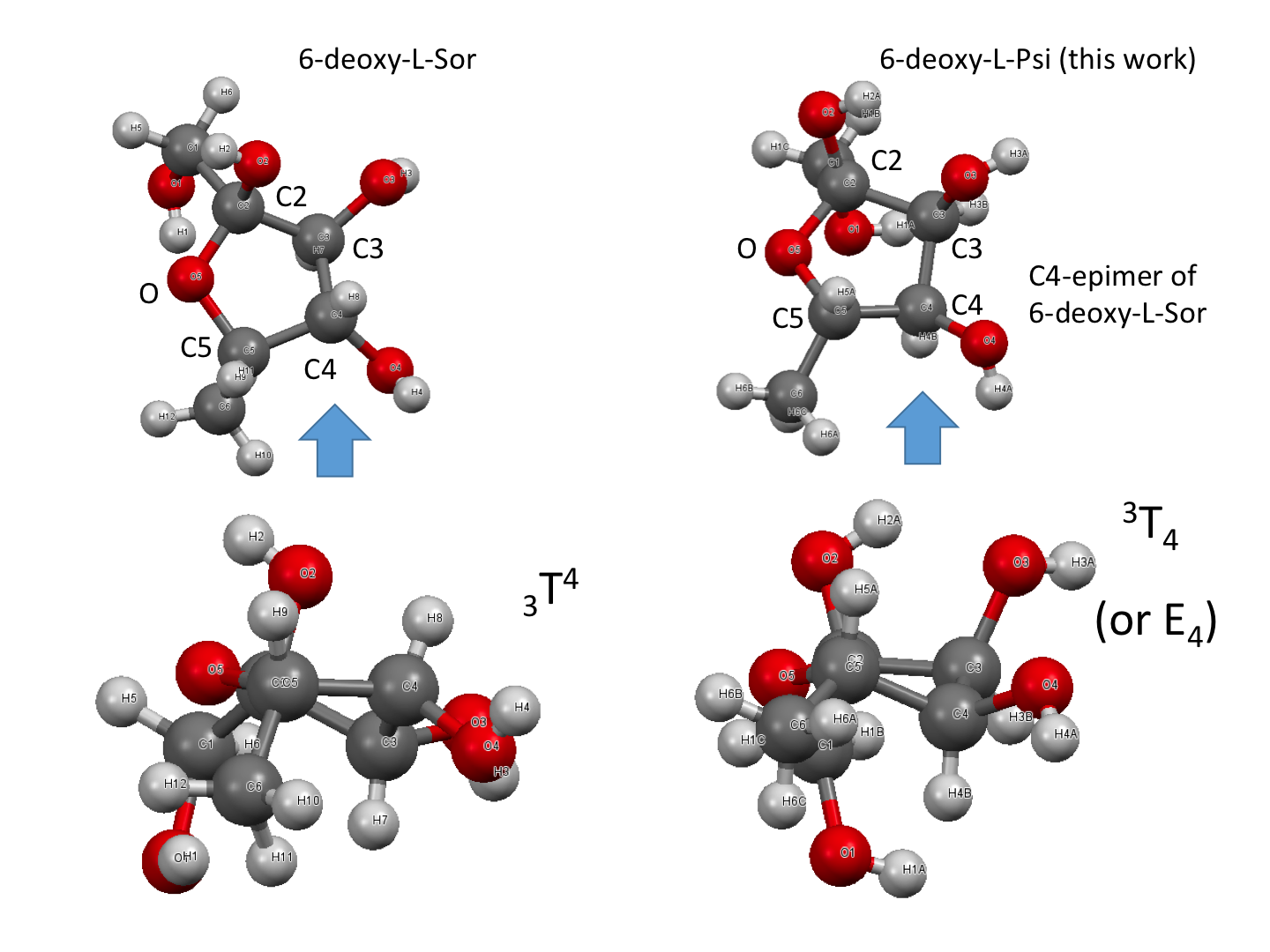

Supplement: Supplementary file 3 [file e-71-0o993-Isup3.png]

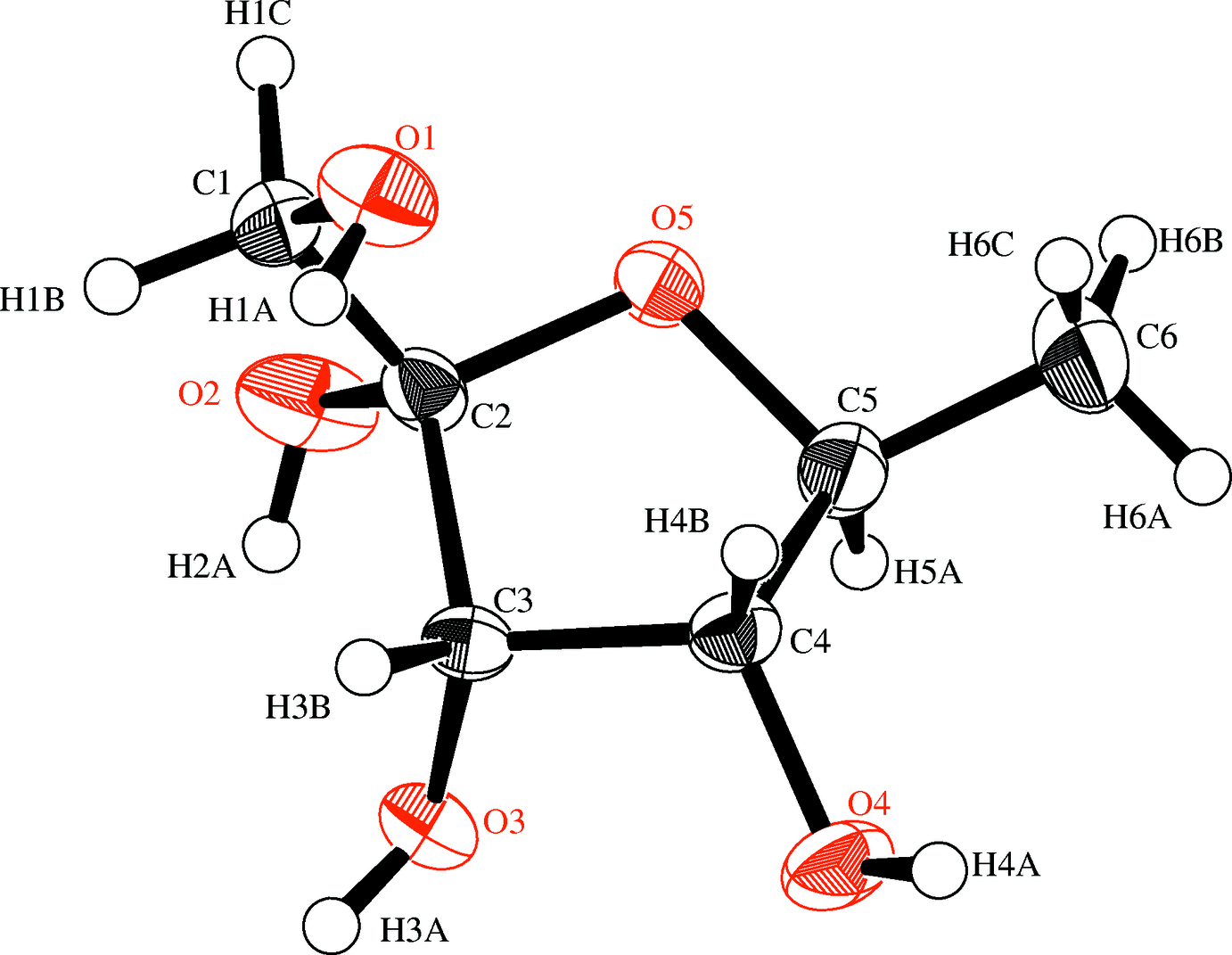

Supplement: Supplementary file 4 [file e-71-0o993-fig1.tif]

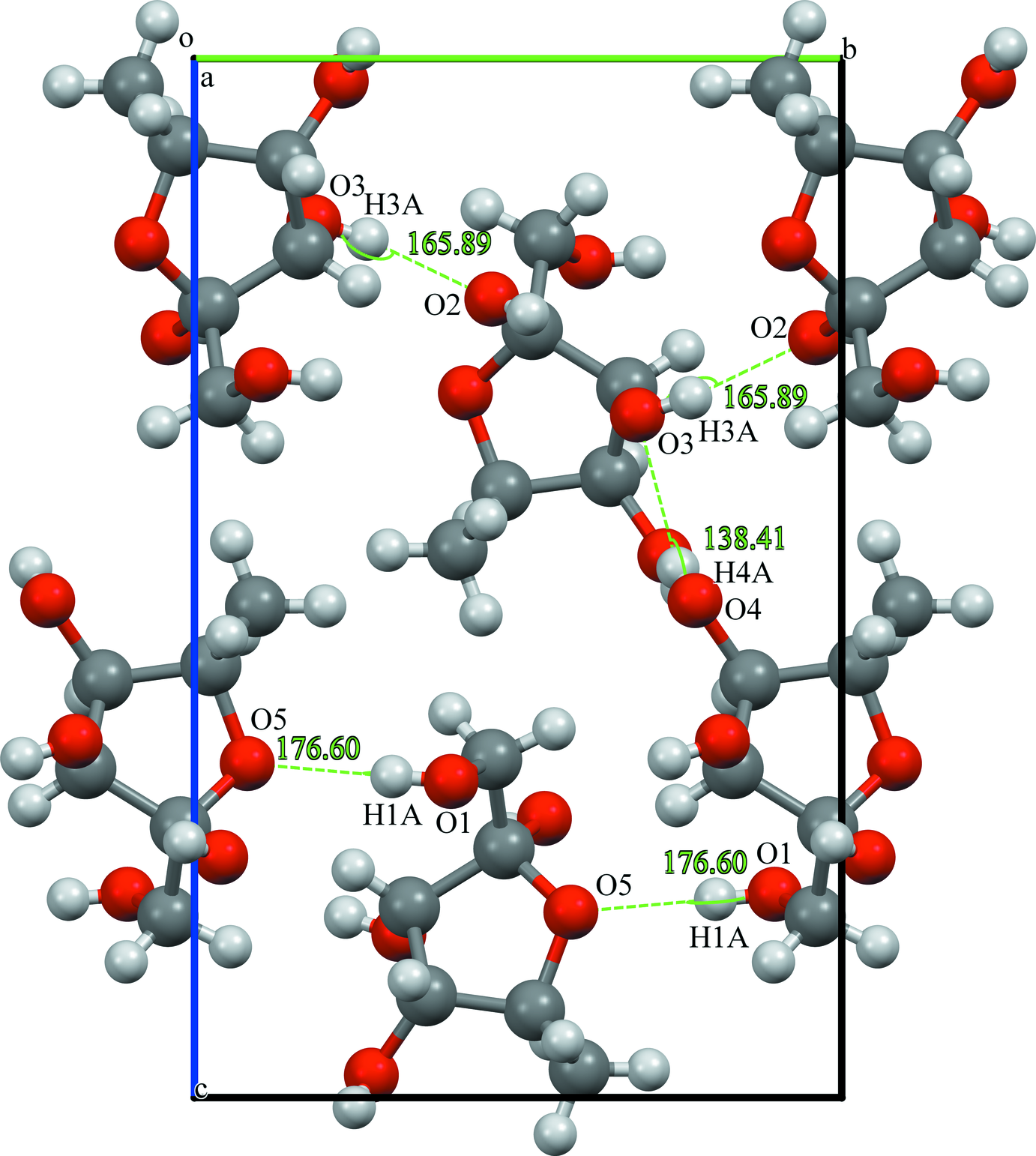

Supplement: Supplementary file 5 [file e-71-0o993-fig2.tif]
